# Somatic mutations in Middle East and North Africa breast cancer patients: a systematic review

**DOI:** 10.1093/oncolo/oyaf205

**Published:** 2025-07-15

**Authors:** Lama Abujamous, Isha Ahmed, Yasmin Ahen, Hadeel Alotaibi, Ala-Eddin Al Moustafa, Shereena Mohd Arif, Hamda Al-Thawadi, Rozami Razali

**Affiliations:** Department of Basic Medical Sciences, College of Medicine, QU Health, Qatar University, Doha, Qatar; Office of Vice President for Research and Graduate Studies, Qatar University, Doha, Qatar; Department of Biomedical Sciences, College of Health Science, QU Health, Qatar University, Doha, Qatar; Department of Biomedical Sciences, College of Health Science, QU Health, Qatar University, Doha, Qatar; Department of Biomedical Sciences, College of Health Science, QU Health, Qatar University, Doha, Qatar; Oncology Department, McGill University, Montreal, Quebec H4A 3J1, Canada; The National University of Malaysia in Qatar, Doha, Qatar; Department of Basic Medical Sciences, College of Medicine, QU Health, Qatar University, Doha, Qatar; Department of Biomedical Sciences, College of Health Science, QU Health, Qatar University, Doha, Qatar

**Keywords:** somatic mutations, breast cancer genetics, PIK3CA, TP53, mutation profiling, personalized oncology

## Abstract

**Background:**

Breast cancer presents with distinct clinical and molecular characteristics in the Middle East and North Africa (MENA) region, where women are diagnosed at younger ages and with more aggressive disease compared to Western populations. Despite the global burden, genomic studies of breast cancer in MENA remain underrepresented. This systematic review provides the first comprehensive analysis of somatic mutations in breast cancer patients across the MENA region.

**Methods:**

Following PRISMA guidelines, we analyzed 44 studies encompassing 13 MENA countries, representing data from over 2500 breast cancer patients. Studies were rigorously assessed using the Newcastle-Ottawa Scale, with mutation data extracted, standardized, and classified according to pathogenicity using established databases. We employed multiple sequencing methodologies, including next-generation sequencing and targeted gene panels, to identify country-specific and region-wide mutation patterns.

**Results:**

We identified 559 mutations across 104 genes, with TP53 (23.79%) and PIK3CA (10.19%) emerging as the most frequently altered genes, followed by significant mutations in BRCA1/2, ATM, ESR1, and PTEN. Nearly 43% of variants were classified as pathogenic/likely pathogenic, while 23% remained variants of uncertain significance. Missense mutations predominated (60.29%), followed by frameshift variants (13.06%) and stop-gained mutations (10.91%). We discovered distinctive country-specific mutation profiles, including unique alterations in KLF6 (Turkey) and IL-1β (Iraq), reflecting potential environmental and hereditary influences unique to MENA populations. Notably, all 11 PIK3CA hotspot mutations that predict sensitivity to alpelisib therapy were identified.

**Conclusions:**

This study reveals both shared and distinct somatic mutation patterns in MENA breast cancer patients compared to Western populations. The high prevalence of clinically actionable mutations, particularly in PIK3CA and DNA repair genes, presents immediate opportunities for implementing targeted therapies across the region. Our findings underscore the urgent need for establishing a MENA Breast Cancer Genomics Consortium to standardize sequencing protocols, develop locally validated gene panels, and create regional variant databases that capture the unique mutation spectrum of these populations. This comprehensive genomic landscape of breast cancer in the MENA region addresses a critical gap in global cancer genomics, ultimately improving outcomes for a historically underrepresented patient population.

Implications for Practice This review highlights the urgent need to integrate routine, panel-based next-generation sequencing into the initial work-up of breast cancer across the MENA. Frequently detecting actionable PIK3CA hotspots and homologous recombination gene defects (BRCA1/2, ATM) supports early identification of patients likely to benefit from PI3K or PARP inhibition, or mutation-directed clinical trials. Establishing a regional somatic-variant database and harmonized reporting framework will reduce the current high proportion of variants of uncertain significance and standardize therapeutic decision-making. Embedding these genomic insights into national cancer control strategies will improve the cost-effective deployment of targeted agents and underpin precision-oncology training for clinicians across the MENA region.

## Introduction 

Breast cancer is the most common cancer in women worldwide and continues to be one of the top causes of cancer related deaths.^1^ Most countries in the Middle East and North Africa (MENA) region share common cultural ties but do exhibits significant diversity in many fields.^2^ According to Najjar and Easson (2010) and Safiri et al. (2022), breast cancer is more common in the younger population in the MENA region in comparison with Western populations and manifests more aggressive characteristics. It is also established that young women’s relative risk of breast cancer is influenced by a number of interrelated factors, such as genetics, lifestyle, and early tumor detection.^3–5^ Numerous classification systems have been developed to divide breast cancer into subtypes based on various biomarkers.^6^^,^^7^ The most widely used breast cancer categorization approach is based on molecular subtyping of biomarkers such as estrogen receptors (ER), progesterone receptors (PR), and human epidermal growth factor 2 (HER2) receptors. According to American Society of Clinical Oncology/College of American Pathologists, breast cancers are classified into four subtypes based on the immunohistochemical expression of HER2, ER, and PR.

Breast cancer can be caused by both germline and somatic genetic alterations.^8^ In contemporary times, the integration of genomic profiling into clinical management of cancer treatment has enhanced the practice of precise oncology.^9–13^ In recent years, majority of the epidemiological studies on breast cancer focuses on germline mutations while disregarding somatic mutations.^14^ In breast cancer, somatic mutations often affect a range of genes, including those involved in tumor suppression, oncogenesis, and DNA repair, with BRCA1/2, TP53, PIK3CA, AKT1, and CDH1 being among the most altered.^15^^,^^16^ Such mutations, especially those involved in oncogenes, tumor suppression, and DNA repair, are pivotal in cancer development, often enabling tumors to grow and resist cell death mechanisms. For instance, mutations that hinder DNA replication or repair can trigger cell cycle arrest and apoptosis.^17^^,^^18^ It is especially important in the MENA region, where environmental and genetic factors may contribute to the development and progression of breast cancer in different ways.

In this study, the aim is to systematically investigate the prevalence of somatic mutations in breast cancer patients from the MENA region. To the best of our knowledge, this is the first systematic review of somatic mutations combining all MENA breast cancer patients.

## Methods

### Search strategy and objectives

The search strategy for this review followed the Preferred Reporting Items for Systematic Review and Meta-Analysis (PRISMA) guidelines.^19^ The MENA definition consist of Qatar, Saudi Arabia, Somalia, Sudan, Syria, Tunisia, United Arab Emirates, Algeria, Bahrain, Djibouti, Egypt, Iraq, Jordan, Kuwait, Lebanon, Libya, Mauritania, Morocco, Oman, Palestine, Yemen, Turkey, and Iran.^20^ PubMed, Google Scholar, Web of Science, Science Direct, ProQuest, Scopus, Embase, and grey literature were searched for studies on somatic breast cancer in 22 MENA countries, from inception until October 2024, using key phrases combined with **“OR”** and **“AND”** between related terms to obtain targeted results for the primary outcome. Keywords included the terms “MENA region,” “Middle East,” “North Africa,” “Somatic mutations,” “Somatic alterations,” “Breast cancer,” “Breast carcinoma,” “Personalized medicine,” “Breast cancer genetics,” “Molecular patterns,” “Molecular signature,” “Precision medicine,” “Biomarkers,” “genetic variation,” “genetic alteration,” “mutations prevalence,” “mutations patterns,” “mutations frequency,” “mutations analysis,” “functional analysis,” “profiling in breast cancer,” “mutations significance,” “mutations spectrum,” “mutations landscape,” “mutations diversity,” “mutations diversity in breast cancer,” “mutations drivers,” “mutations markers,” “mutations characterization,”, “mutations burden,” “mutations sequencing,” “mutations annotation,” “mutations validation”, “epidemiology,” “prognosis,” “therapy response,” “therapeutic target,” “personalized therapy,” “clinical outcomes,” “survival,” “cancer disparities,” “ethnic variations,” “microenvironment,” “treatment resistance,” “targeted therapy,” “Arab countries,” “Middle East,” “Gulf region,” “hormonal receptors,” “HER2,” “BRCA genes,” “TP53 gene,” “PIK3CA gene,” “MAPK pathway,” “EGFR pathway,” “Notch pathway,” “Wnt pathway,” and “Somatic and germline variant in breast cancer,”. The titles and abstracts of all retrieved articles were initially screened, and those meeting the inclusion criteria were further evaluated in detail and included in the final analysis.

### Study selection

**Figure 1.**
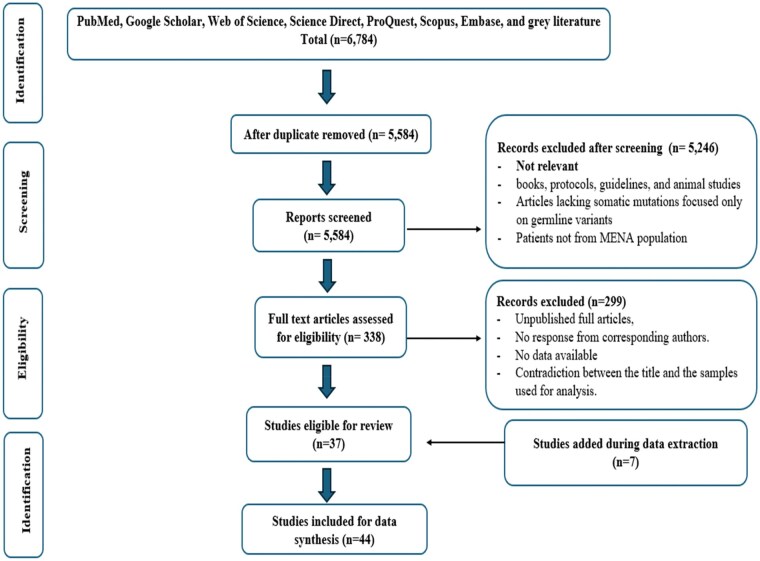
PRISMA flow diagram of the study selection.

We reviewed the retrieved records, resolving discrepancies through consensus with the senior author. The total number of records from each database was documented. Research articles were fully assessed if they met the following inclusion criteria according to the PICOS statement (Table 1): (1) publication in a peer-reviewed journal; (2) population from a MENA country as defined by Michael and Staley;^20^ and (3) focused on breast cancer patients with somatic mutations from formalin-fixed paraffin-embedded (FFPE) or fresh-frozen samples. Articles were excluded if they lacked somatic mutations, focused solely on germline variants from blood samples, unpublished full articles, using restriction fragment length polymorphism (RFLP) or polymerase chain reaction (PCR) without confirmation by sanger method, published abstract without accessible full article, and did not receive a response from corresponding authors, involved a population outside the MENA region, or were books, protocols, or guidelines. After removing duplicates, the remaining articles were reviewed, and records not meeting the inclusion criteria were excluded. Full texts of eligible papers were then assessed to gather relevant information. All assessments, screening and selection process were performed according to PRISMA guidelines (Figure 1).

**Table 1.** Inclusion and exclusion criteria according to the Population, Intervention, Comparator, Outcomes, and Study design (PICOS) statement.

	Included	Excluded
**Population**	MENA countries	Other countries
**Interventions**	None	None
**Comparators**	Breast cancer patients with somatic mutations from FFPE or fresh-frozen samples.	Articles lacking somatic mutations focused only on germline variants from blood samples, unpublished full articles, or with no response from corresponding authors. Using RFLP or PCR without confirmation by the Sanger method.
**Study design**	Prospective, retrospective cohort, case reports, research articles, review, thesis	Books, protocols, guidelines, and animal studies
**Primary Outcome**	Determine the somatic mutations prevalence associated with breast cancer in MENA population	Irrelevant

Abbreviations: FFPE: formalin-fixed paraffin-embedded; MENA: Middle East and North Africa; PCR: polymerase chain reaction; RFLP: restriction fragment length polymorphism.

### Quality control assessment and data extraction

Risk of bias was assessed using the Newcastle-Ottawa Scale (NOS),^21^^,^^22^ which evaluates studies across three domains: Selection (4 points), Comparability (2 points), and Outcome or Exposure (3 points for cohort or case-control studies, respectively), with a maximum score of 9. Studies scoring 7–9 were classified as high quality, 4–6 as moderate, and below 4 as high risk of bias. Higher scores indicate greater methodological rigor and more reliable findings. Data were extracted systematically from both tables and main text of the included studies and organized into a structured spreadsheet. Extracted variables included study title, year, country, methodology, gene names, rsID, COSMIC ID, cDNA and protein changes, mutation effect, and pathogenicity classification. All entries were cross-checked to ensure accuracy.

### Data synthesis and analysis

Given the heterogeneity in study designs, sequencing methods, and reporting standards across MENA countries, a narrative synthesis was conducted in lieu of meta-analysis. Mutation data were categorized by gene, mutation type, country, detection method, and clinical significance, with standardized reporting of gene name, protein change, cDNA notation, and pathogenicity classification. In cases of incomplete annotation, supplemental information was retrieved from NCBI ClinVar, dbSNP, COSMIC, and Varsome to ensure consistency and verification. Frequency distributions were computed for mutation types, gene involvement, and pathogenicity categories. Geographic patterns were assessed by grouping studies by country to identify shared and country-specific mutation profiles. The analysis also tracked methodological progression from targeted single-gene testing to broader next-generation sequencing approaches, emphasizing clinically actionable mutations with therapeutic relevance. Interpretation of regional patterns was informed by the quality of individual studies, with greater analytical weight assigned to those scoring higher on the Newcastle-Ottawa Scale.

### Extended annotation of TP53 and PIK3CA

Since none of the retrieved papers classify variants using the AMP/ASCO/CAP Somatic Variants Guideline, we annotated TP53 and PIK3CA for approved drugs and AMP Classification from OncoKB and Franklin databases, respectively. We selected sequence variants from the two genes that are annotated as pathogenic (from the original papers, ClinVar and Varsome) and manually extracted information from the databases.

## Results

### Search outcome

The review initially covered 22 MENA countries, but nine were excluded due to a lack of accessible studies. From the remaining 13 countries, 6784 records were retrieved and reduced to 5584 after deduplication. Title and abstract screening, followed by evaluation against PICOS criteria, yielded 338 articles for full-text review. Of these, 299 were excluded due to inaccessibility, non-response from authors, missing data, or sample inconsistencies. Ultimately, 37 studies met the inclusion criteria, with 7 additional studies identified during data extraction, resulting in 44 eligible reports. These studies offer a regional overview of somatic mutation profiling in breast cancer, detailing population characteristics, detection methodologies, and frequently reported genes. Direct gene sequencing was the most common method (*n* = 27), followed by targeted gene panels (*n* = 15) and real-time PCR (*n* = 3). Sample sizes ranged from 11 to 280, underscoring the heterogeneity in study design and scale (Supplementary Table 1).

### Quality of the eligible studies

Risk of bias across the 44 included studies comprising cohort, paired comparison, and case-control designs was assessed using the Newcastle-Ottawa Scale (NOS). In the Selection Domain, 43 studies (97.7%) demonstrated adequate representativeness of exposed participants, and all (100%) confirmed the absence of outcomes at baseline. Among case-control studies, 13 of 14 (92.9%) appropriately selected non-exposed participants, with one study (7.1%) indicating potential selection bias. In the Comparability Domain, 38 studies (86.4%) adjusted for primary confounders (e.g., age, gender), while only 30 (68.2%) controlled for additional variables, suggesting that 31.8% lacked adequate confounder adjustment. In the Outcome/Exposure Domain, 27 of 30 cohort studies (90%) employed appropriate exposure measurement, but only 11 (36.7%) justified follow-up duration, and 25 (83.3%) reported follow-up data. Among case-control studies, 10 of 14 (71.4%) adequately ascertained exposure, 11 (78.6%) ensured consistent exposure measurement, but only 5 (35.7%) addressed non-response bias. Overall, cohort studies accounted for the majority (*n* = 30), with case-control studies (*n* = 14) showing a slightly higher mean NOS score (5.43 vs. 5.07), indicating marginally lower risk of bias. Nonetheless, both designs reflect moderate methodological quality (Supplementary Table 2).

### Overall mutation landscape

**Figure 2.**
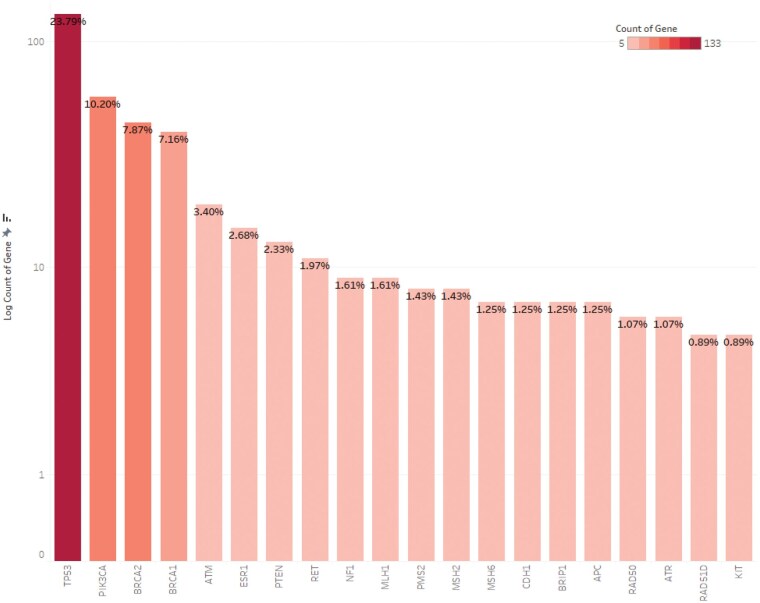
Mutation distribution profile illustrating the prevalence of mutations among the 20 most frequently altered genes in the comprehensive dataset.

**Figure 3.**
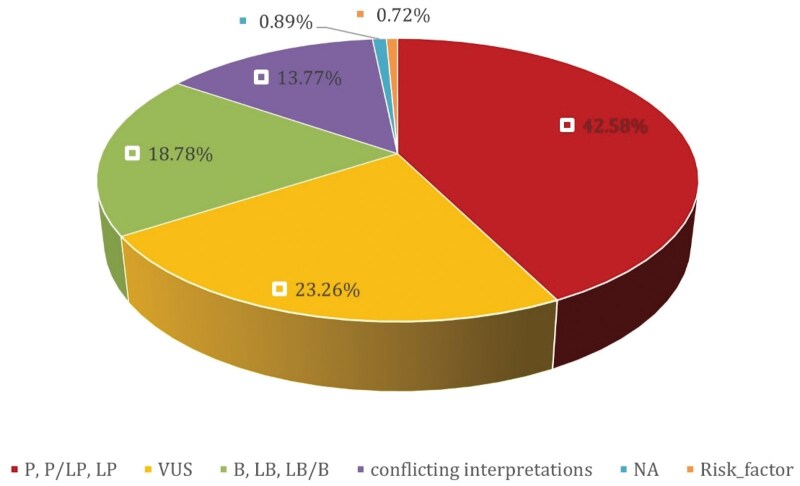
Pie chart for pathogenicity classifications across the analysed dataset.

A total of 698 mutations were identified across the reviewed studies; however, only 559 were included in the final analysis (Supplementary Table 3). The remaining 139 were excluded due to insufficient data for 48 mitochondrial DNA (mtDNA) variants, unclassifiable copy number variants (*n* = 3), inconsistent nomenclature (*n* = 39), and unverifiable transcripts (*n* = 49) due to incomplete details and lack of confirmatory database information. These 559 mutations, derived from patients across 13 MENA countries (Mauritania, Morocco, Tunisia, Egypt, Palestine, Lebanon, Jordan, Iraq, Saudi Arabia, Qatar, Oman, Iran, Turkey), spanned 104 genes. TP53 and PIK3CA were the most frequently analyzed genes (Figure 2), accounting for 23.79% and 10.19% of mutations, respectively. Other commonly reported genes included BRCA1/2, ATM, ESR1, and PTEN, collectively representing 23.43% of variants. While 62.43% of mutations had COSMIC IDs and 83.18% were linked to reference sequences, several lacked entries in ClinVar or other databases, limiting further interpretation. Variant classification revealed that 42.58% were Pathogenic or Likely Pathogenic, 18.78% were Benign or Likely Benign, and 23.26% were Variants of Uncertain Significance (VUS) (Figure 3). Additionally, 0.72% were labeled as Risk Factors, 13.77% showed conflicting interpretations, and 0.89% remained unclassified due to insufficient evidence. Classifications were standardized using contemporary databases as outlined in the Methods. Notably, some variants were reclassified in this study, reflecting updates in the evidence base since their original publication.

**Figure 4.**
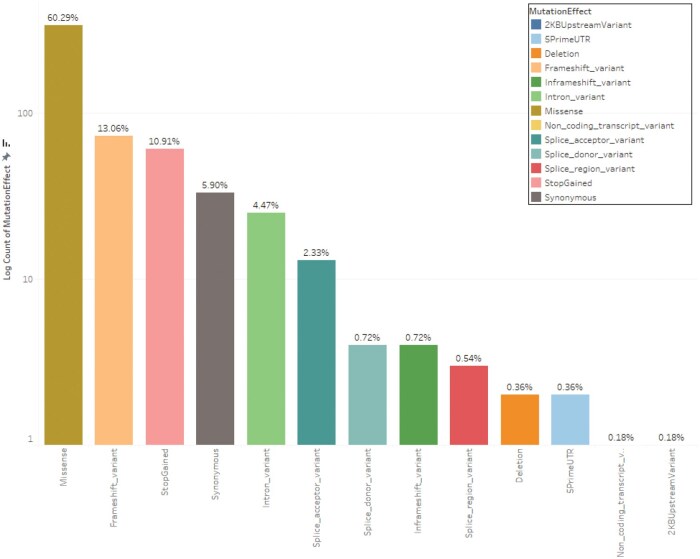
Mutation effect distribution. Pie chart illustrating variant category prevalence (e.g., missense, frameshift, StopGained, etc.) in the genomic dataset, emphasizing dominant types and frequencies.

The predominant mutation types were missense (60.29%), frameshift (13.06%), and stop-gained mutations (10.91%) (Figure 4). Missense variants, resulting from single nucleotide substitutions, frequently disrupt protein function. Frameshift and stop-gained mutations typically produce truncated, nonfunctional proteins. Together, these alterations represent key pathogenic mechanisms in disease progression, warranting further clinical and functional investigation.

### Country-specific mutation patterns

**Figure 5.**
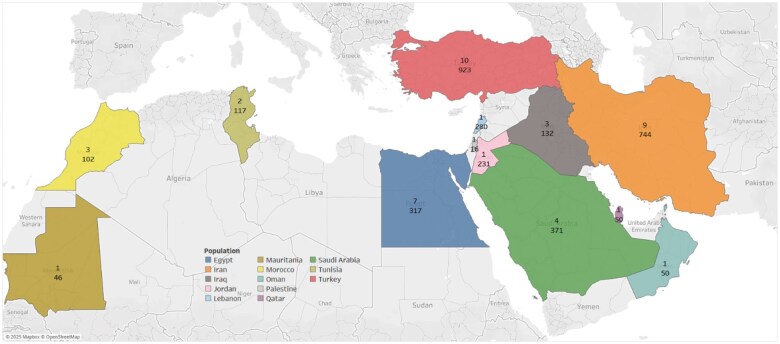
The number of studies (number on top) and the number of samples (number on the bottom) identified across the 13 MENA countries.

Analysis of 44 studies from 13 MENA countries revealed substantial variation in the prevalence of breast cancer-associated genes (Figure 5). In Saudi Arabia, four studies reported 52 genes—the highest among all countries—largely due to the use of broad genetic panels. PIK3CA was the most frequently reported gene, followed by TP53. Turkey similarly exhibited high gene diversity, with 45 genes reported across 10 studies, including TP53, PIK3CA, KLF6, PTEN, and RET. In Egypt, 44 genes were identified, with TP53 and PIK3CA consistently reported, along with BRCA1/2 and ESR1. Morocco reported 11 genes across three studies, predominantly PIK3CA and TP53. In contrast, Iran reported only six genes from nine studies, including unique detections such as mtDNA and ER-α variants. Iraq’s three studies identified IL-1b, VEGF, IL-4, PTEN, and PIK3CA. In Jordan, Lebanon, and Palestine, PIK3CA was the sole reported gene, likely due to limited studies and narrower gene panels. Single studies in Mauritania, Oman, and Qatar reported TP53, BRIP1, and ESR, respectively. A detailed summary of gene distribution by country is provided in Supplementary Table 1.

### Variant interpretation and clinical actionability of key genes

We identified several TP53 and PIK3CA variants classified as Tier 1 under AMP/ASCO/CAP guidelines (Table 2). All TP53 variants carried Level Px1 prognostic evidence, with common alterations such as p. Arg248Gln (rs11540652), p. Arg273His (rs28934576), and p. Arg175His (rs28934578) deemed “Likely Oncogenic” or “Oncogenic” by Franklin and OncoKB. Despite their high pathogenicity, most lacked actionable therapeutic associations in breast cancer, except p. Tyr220Cys (rs121912666), which is linked to Rezatapopt. In contrast, PIK3CA variants exhibited stronger therapeutic relevance. Nearly all were classified as “Oncogenic” or “Likely Oncogenic” with Level 1 evidence. Recurrent mutations such as p. Glu545Lys, p. His1047Arg, and p. Met1043Ile are supported by substantial clinical data and correspond to FDA-approved therapies, including Alpelisib, Fulvestrant, and Capivasertib. Even rarer variants (p.Arg88Gln, rs121913287; p. Arg93Gln, rs106479336) demonstrated actionable potential. These findings reinforce the clinical significance of PIK3CA mutations, particularly in hormone receptor-positive breast cancer, where PI3K pathway inhibition remains a key therapeutic strategy.

**Table 2.** Extended annotation of key genes (TP53 and PIK3CA).

Gene	rsID	cDNA	Protein	AMP classification	Evidence strength	Pathogenicity	Drugs approved
PIK3CA	rs1064793663	c.278G>A	p.Arg93Gln	Tier 1	Level 1/Therapeutic	Likely oncogenic	Inavolisib + Palbociclib + Fulvestrant
PIK3CA	rs121913272	c.1258T>C	p.Cys420Arg	Tier 1	Level 1/Therapeutic	Oncogenic	Alpelisib + Fulvestrant, Alpelisib + Fulvestrant
PIK3CA	rs121913273	c.1624G>A	p.Glu542Lys	Tier 1	Level 1/Therapeutic	Oncogenic	Alpelisib + Fulvestrant, Capivasertib + Fulvestrant
PIK3CA	rs121913279	c.3140A>G	p.His1047Arg	Tier 1	Level 1/Therapeutic	Oncogenic	Alpelisib + Fulvestrant, Capivasertib + Fulvestrant
PIK3CA	rs121913287	c.263G>A	p.Arg88Gln	Tier 1	Level 1/Therapeutic	Oncogenic	Capivasertib + Fulvestrant
PIK3CA	rs2108408396	c.1639G>A	p.Glu547Lys	Tier 2	Likely neutral	Likely neutral	NA
PIK3CA	rs2108429262	c.3010A>G	p.Met1004Val	Tier 1	Level 1/Therapeutic	Likely oncogenic	Inavolisib + Palbociclib + Fulvestrant
PIK3CA	rs104886003	c.1633G>A	p.Glu545Lys	Tier 1	Level 1/Therapeutic	Oncogenic	Alpelisib + Fulvestrant, Capivasertib + Fulvestrant
PIK3CA	rs1057519927	c.1625A>T	p.Glu542Val	Tier 1	Level 1/Therapeutic	Oncogenic	Inavolisib + Palbociclib + Fulvestrant
PIK3CA	rs1057519936	c.3127A>G	p.Met1043Val	Tier 1	Level 1/Therapeutic	Oncogenic	Capivasertib + Fulvestrant
PIK3CA	rs1064793732	c.1093G>A	p.Glu365Lys	Tier 1	Level 1/Therapeutic	Likely oncogenic	Inavolisib + Palbociclib + Fulvestrant
PIK3CA	rs121913274	c.1634A>C	p.Glu545Ala	Tier 1	Level 1/Therapeutic	Oncogenic	Alpelisib + Fulvestrant, Capivasertib + Fulvestrant
PIK3CA	rs121913274	c.1634A>G	p.Glu545Gly	Tier 1	Level 1/Therapeutic	Oncogenic	Alpelisib + Fulvestrant, Capivasertib + Fulvestrant
PIK3CA	rs121913275	c.1635G>T	p.Glu545Asp	Tier 1	Level 1/Therapeutic	Likely oncogenic	Alpelisib + Fulvestrant, Capivasertib + Fulvestrant
PIK3CA	rs121913277	c.3145G>C	p.Gly1049Arg	Tier 1	Level 1/Therapeutic	Oncogenic	Capivasertib + Fulvestrant
PIK3CA	rs121913279	c.3140A>T	p.His1047Leu	Tier 1	Level 1/Therapeutic	Oncogenic	Alpelisib + Fulvestrant, Capivasertib + Fulvestrant
PIK3CA	rs121913281	c.3139C>T	p.His1047Tyr	Tier 1	Level 1/Therapeutic	Oncogenic	Alpelisib + Fulvestrant, Capivasertib + Fulvestrant
PIK3CA	rs121913283	c.3129G>A	p.Met1043Ile	Tier 1	Level 1/Therapeutic	Oncogenic	Capivasertib + Fulvestrant
PIK3CA	rs121913284	c.1035T>A	p.Asn345Lys	Tier 1	Level 1/Therapeutic	Oncogenic	Capivasertib + Fulvestrant
PIK3CA	rs121913285	c.1616C>G	p.Pro539Arg	Tier 1	Level 1/Therapeutic	Oncogenic	Inavolisib + Palbociclib + Fulvestrant
PIK3CA	rs1576949960	c.3132T>A	p.Asn1044Lys	Tier 1	Level 1/Therapeutic	Oncogenic	Inavolisib + Palbociclib + Fulvestrant
PIK3CA	rs1724342112	c.277C>T	p.Arg93Trp	Tier 1	Level 1/Therapeutic	Likely oncogenic	Inavolisib + Palbociclib + Fulvestrant
PIK3CA	rs397517201	c.1637A>C	p.Gln546Pro	Tier 1	Unknown	Likely oncogenic	Capivasertib + Fulvestrant
PIK3CA	rs397517201	c.1637A>G	p.Gln546Arg	Tier 1	Level 1/Therapeutic	Oncogenic	Alpelisib + Fulvestrant, Capivasertib + Fulvestrant
PIK3CA	rs397517202	c.3073A>G	p.Thr1025Ala	Tier 1	Level 1/Therapeutic	Likely oncogenic	Inavolisib + Palbociclib + Fulvestrant
PIK3CA	rs587777790	c.353G>A	p.Gly118Asp	Tier 1	Level 1/Therapeutic	Oncogenic	Inavolisib + Palbociclib + Fulvestrant
TP53	rs2073378475	c.479T>A	p.Met160Lys	Tier 1	Level Px1/Prognostic	Likely oncogenic	_
TP53	rs11540652	c.743G>A	p.Arg248Gln	Tier 1	Level Px1/Prognostic	Likely oncogenic	_
TP53	rs11540654	c.329G>C	p.Arg110Pro	Tier 1	Level Px1/Prognostic	Likely oncogenic	_
TP53	rs11540654	c.329G>T	p.Arg110Leu	Tier 1	Level Px1/Prognostic	Likely oncogenic	_
TP53	rs121912651	c.742C>T	p.Arg248Trp	Tier 1	Level Px1/Prognostic	Likely oncogenic	_
TP53	rs121912656	c.734G>A	p.Gly245Asp	Tier 1	Level Px1/Prognostic	Likely oncogenic	_
TP53	rs121912657	c.814G>A	p.Val272Met	Tier 1	Level Px1/Prognostic	Likely oncogenic	_
TP53	rs121912657	c.814G>T	p.Val272Leu	Tier 1	Level Px1/Prognostic	Likely oncogenic	_
TP53	rs121912660	c.839G>C	p.Arg280Thr	Tier 1	Level Px1/Prognostic	Likely oncogenic	_
TP53	rs121912662	c.1031T>C	p.Leu344Pro	Tier 1	Level Px1/Prognostic	Likely oncogenic	_
TP53	rs121912664	c.1010G>C	p.Arg337Pro	Tier 1	Level Px1/Prognostic	Likely oncogenic	_
TP53	rs121912666	c.659A>G	p.Tyr220Cys	Tier 1	Level Px1/Prognostic & Level 3A/Therapeutic	Oncogenic	Rezatapopt
TP53	rs121913343	c.817C>T	p.Arg273Cys	Tier 1	Level Px1/Prognostic	Likely oncogenic	_
TP53	rs148924904	c.488A>G	p.Tyr163Cys	Tier 1	Level Px1/Prognostic	Likely oncogenic	_
TP53	rs1555526241	c.428T>C	p.Val143Ala	Tier 1	Level Px1/Prognostic	Likely oncogenic	_
TP53	rs17849781	c.832C>T	p.Pro278Ser	Tier 1	Level Px1/Prognostic	Likely oncogenic	_
TP53	rs28934573	c.722C>T	p.Ser241Phe	Tier 1	Level Px1/Prognostic	Likely oncogenic	_
TP53	rs28934574	c.844C>T	p.Arg282Trp	Tier 1	Level Px1/Prognostic	Likely oncogenic	_
TP53	rs28934575	c.733G>A	p.Gly245Ser	Tier 1	Level Px1/Prognostic	Oncogenic	_
TP53	rs28934576	c.818G>A	p.Arg273His	Tier 1	Level Px1/Prognostic	Oncogenic	_
TP53	rs28934578	c.524G>A	p.Arg175His	Tier 1	Level Px1/Prognostic	Oncogenic	_
TP53	rs530941076	c.658T>C	p.Tyr220His	Tier 1	Level Px1/Prognostic	Likely oncogenic	_
TP53	rs55832599	c.799C>T	p.Arg267Trp	Tier 1	Level Px1/Prognostic	Likely oncogenic	_
TP53	rs587780073	c.701A>G	p.Tyr234Cys	Tier 1	Level Px1/Prognostic	Likely oncogenic	_
TP53	rs730882005	c.713G>T	p.Cys238Phe	Tier 1	Level Px1/Prognostic	Likely oncogenic	_
TP53	rs786201059	c.856G>A	p.Glu286Lys	Tier 1	Level Px1/Prognostic	Likely oncogenic	_
TP53	rs786201838	c.578A>G	p.His193Arg	Tier 1	Level Px1/Prognostic	Likely oncogenic	_
TP53	rs786202962	c.527G>A	p.Cys176Tyr	Tier 1	Level Px1/Prognostic	Likely oncogenic	_
TP53	rs786202962	c.527G>C	p.Cys176Ser	Tier 1	Level Px1/Prognostic	Likely oncogenic	_
TP53	rs864622237	c.700T>A	p.Tyr234Asn	Tier 1	Level Px1/Prognostic	Likely oncogenic	_
TP53	rs876658468	c.577C>T	p.His193Tyr	Tier 1	Level Px1/Prognostic	Likely oncogenic	_
TP53	rs876658468	c.577C>A	p.His193Asn	Tier 1	Level Px1/Prognostic	Likely oncogenic	_

## Discussion

### Summary of key findings

Our systematic review analysed 44 studies investigating somatic mutations in breast cancer across 13 countries in the MENA region. The process began with 6,784 articles and resulted in a focused dataset covering mutations in 104 genes. Among these, TP53 and PIK3CA were the most frequently reported, alongside significant findings in BRCA1/2, ATM, ESR1, and PTEN. The mutations were primarily missense variants, frameshift variants and stop-gained mutations. Pathogenic or likely pathogenic mutations made up 42.58% of the dataset, while 23.26% were classified as variants of uncertain significance (VUS).

### Comparison with global mutation patterns

The somatic mutational landscape of breast cancer differs markedly between Western and Arab populations. In Western cohorts, PIK3CA, TP53, and BRCA1/2 are among the most frequently mutated genes. PIK3CA mutations, prevalent in 30%-40% of hormone receptor-positive, HER2-negative breast cancers, are strongly linked to PI3K/AKT/mTOR pathway activation.^23^^,^^24^ Our review reveals lower PIK3CA mutation frequencies in several MENA countries, highlighting regional variation.^25^^,^^26^ Likewise, TP53 mutations, present in ∼20%-35% of global breast cancer cases, displayed distinct mutational spectra in MENA cohorts, including unique alterations reported in Saudi Arabia and Egypt.^25^^,^^26^ These mutations are particularly enriched in triple-negative breast cancers and are associated with aggressive tumor phenotypes.^24^ Notably, elevated rates of APC, PTEN, and KRAS mutations were reported in select Arab subgroups,^6^^,^^27^ while PTEN and RB1 mutations were less frequent in others, underscoring the region’s genetic heterogeneity.^25^^,^^28^ The earlier age of onset and aggressive clinical presentation in MENA patients may, in part, reflect these unique mutational profiles—especially the higher prevalence of alterations in DNA repair pathways. The high prevalence of TP53 mutations (23.79%) in MENA breast cancers is clini­cally significant, given their association with poor prognosis, treatment resistance, and limited therapeutic options.^29^^,^^30^ Although no TP53-targeted therapies are currently approved in breast cancer, emerging trials—such as the Phase II study (NCT03358017) of atorvastatin and zoledronic acid—offer potential by targeting the mevalonate pathway and mutant oncogenes. PIK3CA mutations (10.19%) also represent an actionable target. All 11 hotspot mutations associated with alpelisib sensitivity in the SOLAR-1 and BYLieve trials were identified in our dataset,^31^ suggesting that a subset of HR-positive, HER2-negative MENA patients could benefit from alpelisib–fulvestrant therapy. Their higher frequency in countries like Egypt, Turkey, and Saudi Arabia supports the adoption of routine PIK3CA testing in clinical settings across the region. Beyond these, recurrent mutations in DNA repair genes such as BRCA1/2 and ATM have therapeutic implications for PARP inhibitor use.^32^ The presence of such mutations warrants broader implementation of germline and somatic testing to identify candidates for targeted therapies such as olaparib and talazoparib, which are effective in tumors with homologous recombination deficiency. The VUS inflation (∼23%) reflects the under-representation of MENA genomes in public reference databases and the scarcity of downstream functional validation of region-specific alleles. Functional assays ranging from TP53 trans to CRISPR knockout screens in breast organoids can provide the highest level of evidence (ACMG/AMP PS3/BS3) for upgrading or downgrading a VUS.^33^^,^^34^ Integrating such data can reduces the VUS burden in pivotal cancer gene and directly inform targeted therapy eligibility.

### Country-specific mutation profiles

Regional variation in mutation patterns carries important clinical implications. The identification of unique alterations such as KLF6 in Turkey and IL-1β in Iraq suggests potential for region-specific biomarkers relevant to early detection, prognosis, and therapeutic response. These findings emphasize the need for locally tailored genetic panels, as reliance on panels developed for Western populations may overlook mutations more prevalent in MENA cohorts. Barakeh et al. (2021) examined somatic mutations in 53 breast cancer tumors by sequencing 70 cancer-related genes and reported a high mutational burden, possibly reflecting enhanced genomic instability and tumor aggressiveness.^6^ Their identification of BRCA1, BRCA2, PIK3CA, and TP53 as the most frequently mutated genes aligns with our findings. However, while their data showed high PIK3CA mutation prevalence in Saudi Arabian patients, our broader review revealed considerable variability in PIK3CA frequencies across MENA countries, highlighting intra-regional heterogeneity. Moreover, our review identified significant mutations in DNA repair genes beyond BRCA1/2, notably ATM, which was not prominently reported by Barakeh et al. (2021). Additionally, their observation of lower PTEN and RB1 mutation frequencies in the Saudi cohort is consistent with patterns we observed across multiple Arab populations,^25^^,^^28^ reinforcing the need for population-specific genomic insights.

The observed regional variability in mutation patterns underscores the need for country-specific genomic profiling, rather than assuming genetic homogeneity across the MENA region. Al-Shamsi et al. (2021) reported on the frequency of somatic mutations in Arab women with breast cancer, identifying differences from Western populations and advocating for large-scale epidemiological studies to clarify the prognostic and therapeutic relevance of these mutations.^27^ Although we could not include specific variants from their study due to lack of publicly available data, our findings similarly highlight the high prevalence of TP53 and PIK3CA mutations, consistent with global trends. Interestingly, BRCA1/2 mutations were not reported in their cohort, marking a notable divergence from both global and regional data. Amemiya et al. (2015) identified 59 unique mutations among Saudi Arabian breast cancer patients, focusing on BRCA2, BRCA1, and TP53. However, we excluded this study due to the absence of risk allele data specific to the Saudi population and conflicting variant interpretations when compared to current ClinVar classifications. This reflects a broader challenge in regional genomic research—the lack of standardized variant annotation and interpretation. Similar discrepancies were encountered in other studies, reinforcing the need to cross-validate findings using established databases like ClinVar to ensure accurate and clinically meaningful variant classification. Our regional review also revealed both shared and distinct mutational signatures across MENA countries. In Egypt, TP53, PIK3CA, and BRCA1/2 mutations were detected alongside variants in genes linked to interleukin signaling and hormone receptor pathways, using both NGS and Sanger sequencing. In Iran, PIK3CA emerged as a dominant mutation, reflecting its widespread relevance. Iraqi studies highlighted mutations in PTEN, VEGF, and IL-4, implicating immune and growth factor signaling. Saudi Arabia showed the broadest mutation spectrum, with frequent alterations in TP53, PIK3CA, BRCA1/2, and various DNA repair genes, facilitated by widespread application of NGS technologies. Collectively, these findings illustrate the complex mutational landscape of breast cancer across the MENA region. They emphasize the importance of local genomic data to inform precision oncology strategies, while also contributing to global efforts in understanding population-specific cancer biology.

### Clinical and therapeutic implications

While most countries relied on targeted methods like Sanger sequencing and NGS, some relied on other methods. In Tunisia researcher performed cold PCR, while in Jordan and Lebanon they used real-time PCR adapting their techniques to available resources. The prevalence of PIK3CA mutations in countries like Turkey, Tunisia, and Morocco highlights its pivotal role in breast cancer, aligning with global findings. However, unique regional mutations, such as KLF6 in Turkey and IL-1β in Iraq, reflect the diverse genetic influences shaped by environmental factors and lifestyle. These insights emphasize the need for personalized treatment strategies and encourage greater collaboration and resource-sharing across the MENA region to enhance genetic testing and cancer care.

The analysis reinforces the clinical importance of PIK3CA mutations where almost all of the detected variants were classified as Tier 1 and deemed oncogenic or likely-oncogenic. Notably, mutations such as p. Glu545Lys, p. His1047Arg, and p. Met1043Ile have robust clinical support and are associated with approved therapies, including alpelisib, fulvestrant, and capivasertib. These findings are consistent with the SOLAR-1^35^ and BYLieve^36^ clinical trials, which demonstrated the benefit of alpelisib in hormone receptor-positive, HER2-negative breast cancer harboring PIK3CA mutations. Interestingly, even less common variants such as p. Arg88Gln and p. Arg93Gln showed therapeutic relevance. On the other hand, the PI3K pathway regulates critical cellular functions like proliferation, survival, glucose metabolism, and angiogenesis.^37^ In HR-positive, HER2-negative breast cancer activate PIK3CA mutations, which enhance PI3Kα kinase activity.^38^^,^^39^ Such cases are found in about 40% of patients, primarily in mutational hotspots in exons 8, 10, and 21.^40^ Alpelisib, an oral PIK3CA inhibitor, combined with fulvestrant, is recommended by different guidelines for treating PIK3CA-mutated, HR-positive, HER2-­negative locally advanced or metastatic breast cancer cases, after progression on a CDK4/6 inhibitor and aromatase inhibitor.^35^

In contrast, while TP53 mutations were also frequently observed and classified as Tier 1 with prognostic significance, their therapeutic actionability remains limited in the context of breast cancer. Most TP53 variants, including p. Arg248Gln, p. Arg273His, and p. Arg175His, were identified as likely oncogenic or oncogenic, yet lacked associated targeted therapies. Only one variant, p. Tyr220Cys (rs121912666), was linked to a potential therapy (rezatapopt), which remains investigational. This aligns with current literature indicating that, despite the high prevalence of TP53 mutations—particularly in triple-negative breast cancer—they are not yet directly targetable in clinical practice.^41^ Nevertheless, emerging therapeutic strategies, such as the ongoing Phase II trial (NCT03358017) combining atorvastatin and zoledronic acid with chemotherapy, suggest that indirect targeting of mutp53 and related oncogenic pathways (e.g., YAP/TAZ) may offer future treatment avenues. The idea here would be to use these FDA-approved drugs to block the evolution of the disease by exploiting critical mutagenic factors such as mutp53 and YAP/TAZ. Other therapeutic agents against specific gene mutations in breast cancer also target PIK3CA mutations such as alpelisib, trastuzumab, or pertuzumab for HER2-positive cases.^42^^,^^43^

## Limitations

Several limitations were identified. First, most studies lacked a tiered classification system (diagnostic, prognostic, therapeutic), limiting the clinical interpretability of findings.^33^^,^^34^ Second, variability in gene panels across countries hindered cross-country comparisons and may have led to underreporting of mutations in regions without comprehensive testing. Third, the absence of data from 9 of 22 MENA countries introduces selection bias, potentially skewing results toward countries with stronger research infrastructure. Fourth, inconsistent reporting of clinical-genetic correlations across studies limits conclusions about the prognostic and predictive value of specific mutations. To address these gaps, we advocate for the establishment of a MENA Breast Cancer Genomics Consortium to standardize protocols, develop a shared regional variant database, and create locally validated gene panels tailored to the mutation landscape of MENA populations.

## Conclusion

This is the first systematic review to comprehensively characterize somatic mutations in breast cancer across the MENA region, revealing both globally shared and region-specific alterations. TP53 and PIK3CA emerged as the most prevalent mutations, alongside notable changes in BRCA1/2, ATM, ESR1, and PTEN. Country-specific profiles—such as KLF6 in Turkey and IL-1β in Iraq—suggest distinct genetic backgrounds that may underlie the earlier onset and more aggressive disease observed in MENA populations. The high prevalence of pathogenic variants with therapeutic potential underscores the need for integrating targeted treatments into clinical care. Conversely, the large proportion of variants of uncertain significance highlights a pressing need for region-specific functional studies. Our findings also expose disparities in genomic infrastructure and sequencing methodologies across the region. To address this, we advocate for the creation of a MENA Breast Cancer Genomics Consortium to standardize practices, enhance data sharing, and foster collaborations linking genomic profiles to clinical outcomes. In summary, this review establishes a foundation for precision oncology in MENA breast cancer care while calling for expanded regional investment in genomic research capacity.

## Supplementary Material

oyaf205_Supplementary_Data

## Data Availability

Data are available upon reasonable request by contacting the corresponding author. All data relevant to the study are included in the article.
