# Supplementary material for: Somatic mutations in Middle East and North Africa breast cancer patients: a systematic review
Source: Oncologist. 2025 Jul 15;30(9):oyaf205. doi: 10.1093/oncolo/oyaf205 (PMC12445638; doi:10.1093/oncolo/oyaf205)
Supplement: oyaf205_Supplementary_Data [file oyaf205_supplementary_data.zip › Titles for Supplementary Tables .docx]

 Titles for Supplementary Tables 1, 2, and 3:

1. **Supplementary Table 1:** Characteristics of 44 Regional Studies Profiling Somatic Mutations in Breast Cancer
2. **Supplementary Table 2:** Risk-of-Bias Assessment for 44 Cohort, Paired-Comparison, and Case-Control Studies Using the Newcastle-Ottawa Scale
3. **Supplementary Table 3:** Curated list of 559 breast-cancer–associated mutations across 104 genes reported in patients from 13 MENA countries
